# The Most Effective Interventions for Classification Model Development to Predict Chat Outcomes Based on the Conversation Content in Online Suicide Prevention Chats: Machine Learning Approach

**DOI:** 10.2196/57362

**Published:** 2024-09-26

**Authors:** Salim Salmi, Saskia Mérelle, Renske Gilissen, Rob van der Mei, Sandjai Bhulai

**Affiliations:** 1 Research Department 113 Suicide Prevention Amsterdam Netherlands; 2 Department of Stochastics Centrum Wiskunde & Informatica Amsterdam Netherlands; 3 Department of Mathematics Vrije Universiteit Amsterdam Amsterdam Netherlands

**Keywords:** suicide, suicidality, suicide prevention, helpline, suicide helpline, classification, interpretable AI, explainable AI, conversations, BERT, bidirectional encoder representations from transformers, machine learning, artificial intelligence, large language models, LLM, natural language processing

## Abstract

**Background:**

For the provision of optimal care in a suicide prevention helpline, it is important to know what contributes to positive or negative effects on help seekers. Helplines can often be contacted through text-based chat services, which produce large amounts of text data for use in large-scale analysis.

**Objective:**

We trained a machine learning classification model to predict chat outcomes based on the content of the chat conversations in suicide helplines and identified the counsellor utterances that had the most impact on its outputs.

**Methods:**

From August 2021 until January 2023, help seekers (N=6903) scored themselves on factors known to be associated with suicidality (eg, hopelessness, feeling entrapped, will to live) before and after a chat conversation with the suicide prevention helpline in the Netherlands (113 Suicide Prevention). Machine learning text analysis was used to predict help seeker scores on these factors. Using 2 approaches for interpreting machine learning models, we identified text messages from helpers in a chat that contributed the most to the prediction of the model.

**Results:**

According to the machine learning model, helpers’ positive affirmations and expressing involvement contributed to improved scores of the help seekers. Use of macros and ending the chat prematurely due to the help seeker being in an unsafe situation had negative effects on help seekers.

**Conclusions:**

This study reveals insights for improving helpline chats, emphasizing the value of an evocative style with questions, positive affirmations, and practical advice. It also underscores the potential of machine learning in helpline chat analysis.

## Introduction

### Driving Factors for This Study

Worldwide, helplines have been set up to answer thousands of people with suicidal thoughts every day. With technology advancing and the internet having become a big presence in daily life, helplines can now often also be contacted online through chat services. An important question that is yet to be answered regarding helplines is what counselling approach is effective to take. Helplines are often anonymous, thereby making it difficult for evidence-based research, and little is still known. Several studies have been conducted on the Crisis Text Line to identify the characteristics of help seekers and their perception of the helpline’s effectiveness [1,2]. Furthermore, Gould et al [3] examined call reports of help seekers calling helplines in the National Suicide Prevention Lifeline network. In studies by Mokkenstorm et al [4] and Mishara et al [5], helpline chat logs were annotated and analyzed for gathering empirical evidence. A downside of these approaches is that manual annotation of chat logs is often time-consuming work, and not a lot of available data are left unused.

In recent years, significant advancements have been made in the field of natural language analysis. Deep learning models such as transformers have enabled more effective use of big data [6]. Furthermore, bidirectional encoder representations from transformers (BERT) models [7] have made transfer learning a more viable practice. These models provide an opportunity to perform a large-scale analysis of helpline chat data. By training a model and using interpretation methods to view salient conversation features, it is possible to receive an indication of what this model thinks is important to do or not to do in a helpline chat conversation. This can be used to possibly support current findings or lead to new insights.

113 Suicide Prevention, the national suicide prevention helpline in the Netherlands, uses prechat and postchat conversation questionnaires to assess the help seeker’s mental well-being [8], that is, questions related to associated suicide risk factors such as hopelessness, entrapment, perceived burdensomeness, and thwarted belongingness. In this study, we used these risk factor data as an indication of the positive outcome of the intervention. In this way, we were able to gather large amounts of labeled data without the need for manual annotation. From these data, a classification model could be trained to predict chat outcomes based on the content of the chat conversation. We assumed a lower score indicated that the help seeker was less distressed, and therefore, the conversation was labeled as positive and negative otherwise. A better understanding of what contributes to a positive conversation could help inform helplines and possibly result in actionable recommendations for helpline policy.

However, this approach requires addressing 2 main challenges. First, similar to the sentiment analysis of large documents, a decent level of accuracy can be difficult to achieve [9]. The main limitations of transformers are very long-range dependencies because self-attention scales quadratically in the length of the sequence. The *O* (n^2^) time and memory complexity means that the text must be truncated before it can be encoded by the model, and truncation means information will be lost. The long length of the conversations in a crisis line easily leads to a lot of loss of information. Second, the model should be interpretable, such that insights can be gained from the relation of the text content to the classification, that is, which parts of the conversation have more impact on the model’s output.

Both challenges can be addressed using hierarchical models [10,11]. By first passing a subset of the sequence through the model, a representation for that subset can be learned. A text-based chat conversation can quite easily be segmented into individual messages or groups of concurrent messages to use as the first level in the hierarchy. At a second level of hierarchy, another sequence-based model can use the input of the first level to produce the final prediction.

In the domain of text analysis for health care, several applications of transformers have been used to gain insights into health care text data. Gao et al [12] found that pretrained BERT models did not outperform simpler methods for medical document classification. The simpler methods consisted of a convolutional neural network and a hierarchical self-attention network, which had similar performance while having fewer learnable parameters. Ilias and Askounis [13] used local interpretable model-agnostic explanations (LIME) to find influential words of BERT classifications of dementia transcripts.

In this research, we trained and compared a hierarchical document classification approach with the goal of gaining insights into the quality of helpline text-based chat conversations. The first level of the hierarchy leverages pretrained BERT network to obtain embedding representations of chat messages. We theorized that a shallow second level, for example, the message level, would allow for an easier extraction of salient chat messages for interpretation. We tested several different approaches for the message level encoding: a baseline mean pooling layer, a weighted average model with conversation participant masking, a long short-term memory (LSTM) model [10], and a transformer encoder model [10,11]. We compared these models to 3 models that did not use a hierarchical approach. Afterward, we ranked salient messages for improved and not improved scores after helpline conversations by using the best performing model. The salient chat messages where then labeled based on motivational interviewing concepts by 2 experts from the helpline.

### Background

Many state-of-the-art language models that have been developed in recent years rely on transformers. First introduced by Vaswani et al [6], transformers leverage the self-attention mechanism to create long-range connections in a sequential input. This mechanism uses scaled dot-product attention (equation 1).

Attention (*Q,K,V*) = softmax (QK^T^/√d_k_)*V* **(1)**

The projections of the input sequence *Q* and *K* are used to compute a weighted average of the final projection *V*. To prevent the weights from getting too large, in turn causing the gradients to become too small, they are scaled by the dimension *d_k_* of the input sequence. In the original paper, the transformer was developed as an encoder-decoder network for the task of language translation. Devlin et al [7] adapted the encoder section of the transformer to create high-quality embeddings. Dubbed BERT, this network, or variants thereof were applied to obtain state-of-the-art performance on many natural language processing tasks [14]. Many pretrained variants of these networks have been made available. For Dutch language tasks, there are 2 variants trained: Bertje, based on the BERT network and RobBERT, based on the RoBERTa network. Due to the nature of the attention mechanism, a straightforward assumption to make is that the attention weights directly relate to token importance. However, this assumption has been frequently questioned. Serrano and Smith [15] found that attention weights only noisily predict importance.

Jain and Wallace [16] argue attention weights do not provide explanations, while Wiegreffe and Pinter [17] in turn challenged their claims. They argue that there is a time and a place for it and provided tests to determine when attention can be used in such a way. A frequently suggested alternative to attention weight as an importance metric is gradient-based saliency [18,19]. However, even saliency maps have limitations [19]. LIME [20] is a general method for explainability that can also be applied to natural language processing. LIME generates explanations for complex models by locally approximating their behavior with simpler models.

Several adaptations of the transformer method have been proposed to deal with the issue of classification of long documents. Longformer got around the *O (n^2^)* time and memory complexity by using windowed attention, combined with a limited number of global task-specific tokens [21]. An alternate approach is used with hierarchical networks [9,11]. By first computing a fixed representation for a smaller section of the sequence, these representations are used as input for another sequence-based approach. In this method, a sequence has to be split up in some way. Often, paragraphs are used as the delimiter; however, in the case of conversations, a message or utterance could also be appropriate. Lu et al [11] used a hierarchical BERT classification method to also extract salient sentences from documents for improved explainability of the model decisions. In this paper, we use this concept for helpline conversations. As we previously mentioned, saliency from model parameters is a contested approach in literature. Because of this, we included a weighted average approach in our comparison of the hierarchical models as a shallow alternative.

## Methods

### Task Definition

We modelled the problem of predicting the outcome of a text-based helpline chat conversation as a binary classification task. We compared the scores of the questionnaire before and after the conversation. The classification outcome was defined as whether the help seeker’s score on the questionnaire for suicide risk factors improved or did not improve.

### Data Collection

The data consisted of chat conversations of a suicide prevention helpline. Between August 2021 and January 2023, help seekers (N=6903) of this helpline were asked to fill in a short questionnaire on suicide risk factors before and after the conversation with a counsellor. Table 1 [22-28] lists all the items in the questionnaire. To find the class for each conversation from the questionnaire output, we performed the following: we summed the scores of all the items for the prechat and postchat questionnaires. If the summed score for the postchat questionnaire is strictly lower than that of the prechat questionnaire, then we label a conversation as improved, and we label a conversation as not improved otherwise. Conversations that already started at the best possible value for the questionnaire before having the conversation were left out of the data set. Conversations in the suicide prevention helpline also included a triage, where the help seeker was screened for safety. The triage part of the conversation was left out of the data set as well. Without the triage, the average number of messages per conversation was 64.64 (SD 34.81). Due to a large class imbalance between improved and not improved pre-post scores for conversations, we rebalanced the data. Randomly, samples from the larger positive class were removed so that it matched the size of the negative class. The resulting initial data set consisted of 6000 chat conversations. We used an 80:20 split for train and validation sets. We used 903 conversations as the test set, which were obtained during training and validating. This test set was also used for the explainability approaches.

**Table 1:** Items of the prechat and postchat questionnaires.

Variable	Item	Reference
Suicidal ideation	I feel the urge to kill myself	[22]
Unbearable psychache	I can’t take my pain anymore	[23]
Hopelessness	I feel hopeless	[24]
Defeat	I feel that I have given up	[25]
Entrapment	I feel trapped	[26]
Perceived burdensomeness	I am a burden to others	[24]
Thwarted belongingness	I feel like I do not belong	[27]
Desire to live	I have the desire to live	[24]
Capability for suicide	I could kill myself if I wanted to	[28]

### Hierarchical Approach

The individual messages were embedded using a pretrained RoBERTa network called RobBERT [29]. This network was subsequently fine-tuned on the chat conversations by using a triplet-loss strategy. The models that are described in the remainder of this section used this network to embed individual chat messages first. A message embedding was created using a mean pooling layer, resulting in a matrix *M* of dimension *b × l × d*, where *l* is the length of the longest sequence, *d* the embedding size of the pretrained network, and *b* the batch size.

**Figure 1:**
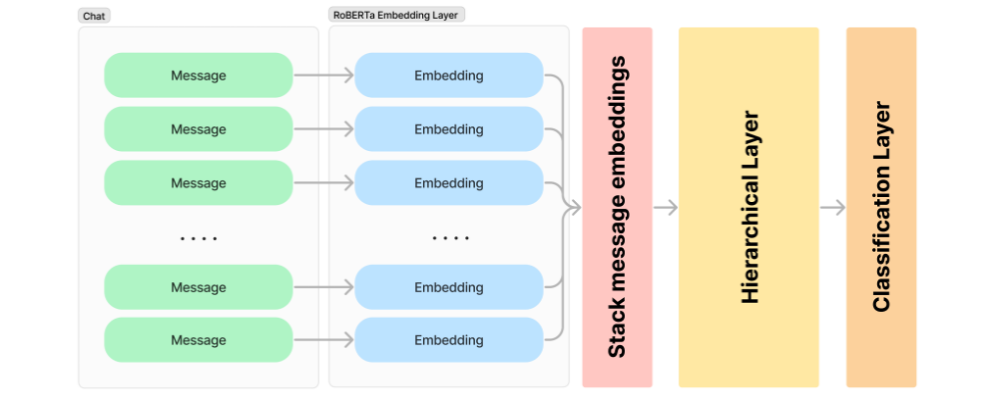
Overview of the hierarchical classification of chats.

The message embeddings for each conversation were then stacked for the hierarchical step. In the hierarchical step, we convert *M* to matrix *C* of dimension *n* × *d*, which is then used in the final binary classification layer. Figure 1 shows a visual overview of this hierarchical approach.

### Weighted Average

To improve explainability, we used a simpler adaptation of the attention mechanism. The weighted average is described in equation 2.

*Weighted average (C) = softmax ((CW_k_^T^ + b_k_)^T^)(CW_v_^T^ + b_v_)* **(2)**

Here, *C* is of dimension *n* × *d,* where *n* is the number of messages and *d* the embedding size. *W_k_* and *W_v_* are learnable weight matrices of dimensions *1* × *d* and *d* × *d*, respectively. This approach can also be described as a simplified version of dot product attention, where only a single class token attends to the sequence. This removes the need for the projections *Q* and *K*. This weighted average results in a *d* dimensional vector, which is used as input for the final feedforward layer for classification. Because we were also interested in the speech of the counsellor in particular, 1 additional adaptation we made was the inclusion of participant masking. Each weighted average is conditional on the sender. So, in a conversation, each weighted average only considered the messages of each participant. This was done by using multiple weighted averages and masking the logits of the weights for the weighted average, which corresponded to each participant. As is common in transformer models, we also used multiple heads, which meant the model created multiple weighted averages. The final heads were then concatenated and projected to a classification output.

Before the message embeddings were combined into the weighted average, the weights were first masked. We created 2 masks: one for only the counsellor message and one for only the help seeker messages. This resulted in the weighted average only being an average of either the counsellor or help seeker. The counsellor and help seeker each had the same number of heads.

### Other Hierarchical Models

We also applied the same hierarchical method of embedding the chat messages and hierarchically classifying these shorter inputs with 3 other methods. We applied a 4-layer LSTM [30] on message embeddings. We also applied 4 transformer embedding layers. A trainable class vector was concatenated to each sequence, which was pooled as the output. The final method applied a simple average of all message embeddings over the sequence dimension. The outputs of these models were fed in the same feedforward layer as the weighted average method.

### Baseline Models

We applied several additional preprocessing steps for the baseline models. All words were also lowercased, lemmatized, and all special characters and punctuations were removed as well as stop words. During tokenization, we limited the number of tokens to 2000. We vectorized the chat conversations by using TF-IDF (term frequency–inverse document frequency) [31]. Finally, each embedded conversation was trained on a support vector machine [32]. Furthermore, the Dutch BERT model, RobBERT, was used as another baseline model. Because it has a maximum length of 512 tokens for the text input, the chats were truncated at the maximum length. Two RobBERT models were fine-tuned—one where the start of the conversation was truncated and one where the end was truncated.

### Explainability

To gain insights into the workings of the network, we employed 2 techniques. First, we used the weights of the weighted average model. The assumption was that messages with a higher weight were of higher importance to the final decision and therefore, more important to the result of the conversation. As a second technique, we applied LIME [20] to the models. For this approach, we left out counsellor messages one at a time to compute the difference in loss. A larger difference indicates more importance to the classification.

The process was as follows: first, we did a feedforward pass of the chat conversations in the test set through the model. Second, we recorded the resulting logits. We selected the chats with a logit score of less than 0.2 and higher than 0.8 that were also correctly classified. Third, we used LIME on just the hierarchical part of the model (yellow part in Figure 1), where each message is a feature, to obtain feature importance scores for each message. We also recorded the weights W_k_ from the weighted average approach. We then selected all the messages that were at least 1 SD above the mean as impactful messages for each of the 2 explainability methods. Each chat that was selected in step 2 was annotated with which the messages were deemed important by the model. We then prompted 2 clinical psychologists operating in the helpline. As this approach is exploratory, we did not formulate a hypothesized set of possible behaviors beforehand for the experts to choose from. However, the 2 experts were trained in motivational interviewing, which was the main paradigm used in the helpline for the chat conversations [8]. Through this lens, they were asked to annotate each impactful message and indicate the behavior conform to this method. From these labels, the most frequently occurring labels were compiled.

### Ethics Approval

This study protocol was performed in accordance with the relevant guidelines. This study was reviewed and approved by the Medical Research Ethics Committee of Amsterdam Universitair Medische Centra (registration: 2021.0447).

## Results

### Model Performance

Table 2 shows the performance scores on a held-out test set. The hierarchical weighted average model was the best performing model with an accuracy of 0.683 and the highest *F*_1_*-*score of 0.688. This was closely followed by the hierarchical LSTM model, which had an accuracy of 0.672. The results for the hierarchical transformer model and hierarchical average did not perform as well, with accuracies of 0.638 and 0.640, respectively. The support vector machine model had an accuracy of 0.638, which was lower than that of the hierarchical models. The 2 truncated versions of the BERT model had accuracies of 0.570 and 0.629 for the truncated end and truncated start models, respectively. This suggests that the information in the truncated text was most likely insufficient compared to the hierarchical models that do not have the ability to attend to words from different messages, but overall have more information available. Overall, our results suggest that the hierarchical LSTM and hierarchical weighted average outperformed other models for the task classifying suicide helpline chat conversations.

**Table 2:** Model performance on the test set of the suicide chat classification task.

Model	Accuracy	Precision	Recall	*F*_1_*-*score
Support vector machine	0.638	0.635	0.632	0.633
BERT^a^ truncated end	0.570	0.556	0.699	0.620
BERT truncated start	0.629	0.605	0.743	0.667
Hierarchical average	0.640	0.621	0.721	0.667
Hierarchical weighted average	0.683	0.679	0.697	0.688
Hierarchical LSTM^b^	0.672	0.674	0.668	0.671
Hierarchical transformer	0.638	0.612	0.754	0.676

^a^BERT: bidirectional encoder representations from transformers.

^b^LSTM: long short-term memory.

### Model Explanations

**Figure 2:**
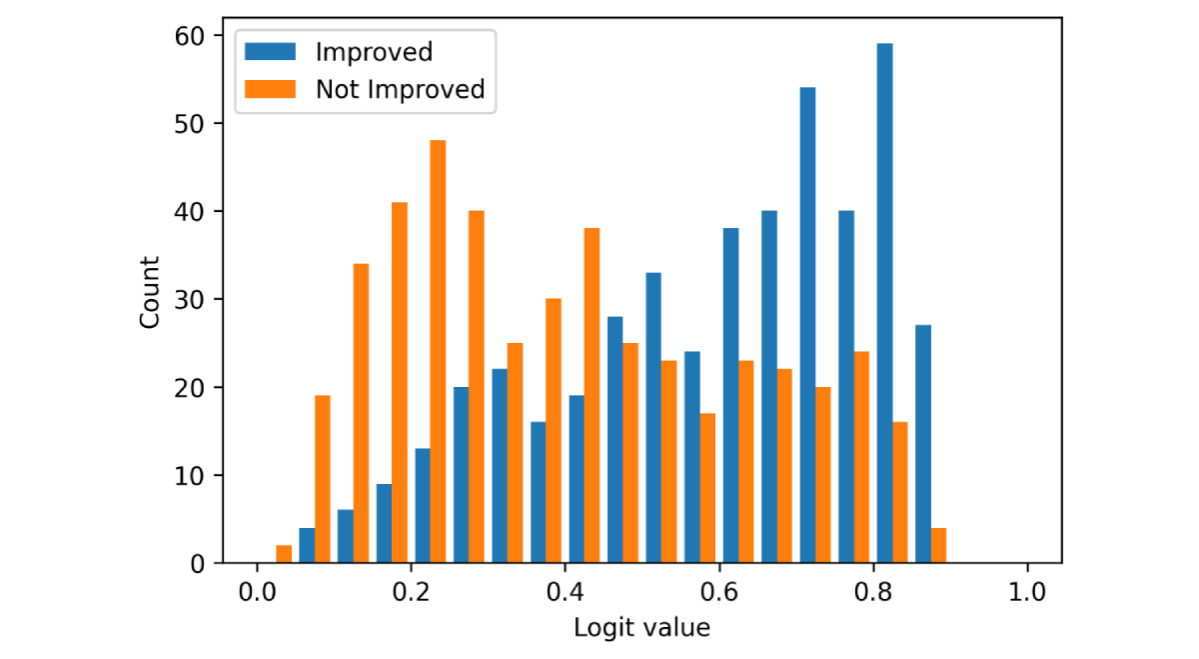
Histogram of logits for the hierarchical weighted model.

The weighted average model was overall the best performer in terms of accuracy and *F*_1_*-*score. Because it was more interpretable than the hierarchical LSTM and the BERT networks, it was the obvious choice to extract explanations. The explanations were compiled from a test set only, and a subselection of the data was made, with only the correctly classified samples and where the model was confident in its output. This confidence was measured through the logit output of the model. A logit value close to 1 corresponds to the classification of a chat conversation that resulted in an improved score and closer to 0 for the opposite case. Figure 2 shows a histogram of the logit outputs of the model for this test data set. Two peaks can be seen for the correctly classified samples. This shows that while there are still chats that are difficult to categorize, there is a clear set of chats where the model is confident for either class. We can also see that the model was slightly more confident for the not-improved chat conversation than for improved ones. We chose values below 0.2 and above 0.8 as subsets to extract the explanations.

Using the weights from the hierarchical weighted average model, we compiled the most influential messages from the counselors. The messages selected as influential were messages with a weight 1 SD above the mean. The weights used for this purpose were from the heads that were masked for the help seeker and thus only contained nonzero values for counselor messages. Furthermore, LIME was used in combination with the weighted average model to obtain explanations. This section describes the outcomes based on observations by the authors and 2 senior psychologists from the helpline.

### Conversations Without an Improved Suicide Risk Score

For conversations that did not improve, we identified 3 distinct situations that emerged from the influential messages. The first and the most common situation was when a conversation ended prematurely. In such cases, the counselor would typically try to redirect the help seeker to alternative channels for assistance, such as a general practitioner, a different helpline, or emergency services. Alternatively, in some cases, the counselor suggested to contact the helpline at another time or to apply for a web-based therapy service. The second situation involved messages where the counselor was unable to respond promptly to the help seeker due to a high volume of ongoing conversations. The counselor apologized to the help seeker for the delay and sometimes mentioned that the helpline was particularly busy and that the counselor was dealing with multiple help seekers simultaneously. The third situation included the counsellor not connecting properly with the help seeker. This was expressed in the use of macros and lists. The macros would often include a standardized set of options for the help seeker to consider or sometimes a set of websites and resources to visit. Sometimes this was also expressed as the counsellor not properly listening to the help seeker. Table 3 shows examples of 5 messages from conversations that did not improve the score on the postchat questionnaire.

**Table 3:** Messages that were the most influential according to the model in the 5 conversations for which the model was the most confident that the conversation had not improved the score on the postchat questionnaire.

Message	Details
Example 1	…*Okay help seeker. To cope with these thoughts at this moment, there are a few options: seeking distraction, contacting someone in your environment, expressing emotions in a pleasant way (creatively, through sports, writing, etc), and relaxation exercises (eg, mindfulness, breathing exercises).*
Example 2	…*At [url], there are tips on how to handle moments like these. Maybe you can take a look to see if there's something that feels right for you to do now?*
Example 3	…*Our chat is exclusively for people with suicidal thoughts. I suggest you contact other helplines if you want to talk about what's on your mind now.*
Example 4	…*That's not the case, help seeker. That confusion must be troubling and occupying your thoughts. I wish I had an answer for you, but I don't have one right away. I notice that you are sharing more, so perhaps it's a good idea to contact the listening line. I wish you a lot of strength, help seeker!*
Example 5	…*I notice that I'm not quite sure what you would like to discuss during this conversation. I want to help you, but I don't really know what you want to talk about.*

### Conversations With an Improved Suicide Risk Score

The salient messages from conversations with improved scores had a wider range of responses compared to those that did not improve scores. However, we identified 2 frequently recurring situations in conversations that showed an improved score. In the first situation, the counselor provided positive reinforcement to the help seeker. During these conversations, the counselor would typically use supportive language, such as showing empathy, offering praise, and expressing happiness for the help seeker. In the second situation, the counselor expressed involvement. For example, the counselor would think along with the help seeker and provide concrete solutions to the help seeker. These solutions could include specific actions or resources tailored to the help seeker’s individual situation. The counselor would provide the help seeker with practical steps that could be taken or resources specific to the help seeker’s situations. Lastly, the 2 less often recurring situations were situations where the counsellor would ask open-ended questions as well as show respect for the autonomy of the help seeker by asking what they wanted to do. Table 4 shows examples of 5 messages from conversations that improved the score on the postchat questionnaire.

**Table 4:** Messages that were the most influential according to the model in the 5 conversations for which the model was the most confident that the conversation improved the score on the postchat questionnaire.

Message	Details
Example 1	…*I find it incredibly admirable that you have clarified this for yourself and that you are going to discuss it with your therapist.*
Example 2	…*You're amazing for taking this on right away.*
Example 3	…*Okay, it's good that you want to try, help seeker. Can I assist you further with something else, or is there something specific you'd like to talk about?*
Example 4	…*I see that you mentioned you are not in treatment. You said that a lot has happened over the past few months that is troubling you and that you tried to drown yourself tonight. I think it’s important that you talk to someone about this so you can get the help you need because you deserve it, dear help seeker.*
Example 5	…*Are you familiar with relaxation exercises? These can usually help with panic attacks as well.*

## Discussion

### Principal Findings

This study compares the performance of different models for classifying suicide helpline chat conversations and found that the hierarchical weighted average model had the best performance. This study also extracted explanations from the hierarchical weighted average model and identified 3 distinct situations for conversations that did not improve and 2 clear recurring situations for conversations that showed an improved score. The results showed that the model had an easier time determining when a conversation would not lead to an improvement in the risk factors. This was also apparent in the explanations where clear and easy distinctions in the output could be made, whereas this was not as easy to do in the case of positive examples.

The research by Mishara et al [5] found that collaborative problem-solving significantly predicted positive outcomes in helpline calls. In line with these findings, our study shows that messages with positive reinforcement and concrete solutions contributed to positive outcomes in chat conversations. Furthermore, Côté and Mishara [33] found through qualitative analysis that reinforcing a strength or a positive action was a significant predictor for increased scores on a pre-post questionnaire in a text message helpline setting. This is in line with our finding that positive reinforcement was a frequently occurring impactful message. In a qualitative study, Gilat and Rosenau [34] analyzed volunteers’ perspective of effective methods in helpline conversations. Among their findings, they identified practical advice as an effective strategy. Building rapport was another aspect of note that their study identified. Because building rapport is highly specific to the individual, this might be something our method was not able to generalize and pick up. However, positive reinforcement could also have been a contributor to building rapport. Overall, these findings highlight the potential of using machine learning models to analyze suicide helpline chat conversations and provide insights into the most influential messages. This allows helplines to be more informed and possibly enable them to improve helpline quality.

### Limitations

Although this study sheds light on influential messages in suicide chat conversations, there are 3 key limitations to be considered. First, there are general limitations of machine learning. The classification task was found to be difficult, as indicated by the 68% accuracy rate that was achieved. This suggests that the current models have room for improvement. There might still be relationships that the current models were not able to capture. It could also be that there is considerable noise in the data set because the outcome measures were self-reported by help seekers, which might not be equally reliable for every help seeker. Furthermore, the indicated influential messages might be messages that are not the main cause but rather a result of a different action. For example, we saw multiple situations where the counsellor expressed gratitude for a compliment. This was most likely the result of the help seeker being grateful for something; however, it does not necessarily mention what the help seeker was grateful for. Second, a limitation of this study is the challenge posed by modeling a large amount of text. Current methods have limitations in capturing dependencies over long ranges or in exceeding maximum memory thresholds, which was the case with the chat conversations used in our data set. Therefore, hierarchical models were used, which had the limitation that dependencies between words from different messages were not captured. Third, a limitation of using chat messages as output to determine categories of influential messages is the need for human judgement. This introduces subjectivity and the potential for bias, as different judges may interpret the same messages differently or possibly miss a connection between the different messages.

### Future Work

Considering the findings presented in this study, we identified 3 potential directions for future work that could further enhance the classification and identification of influential messages in suicide helpline chat conversations. First, while larger models have the potential to improve performance, explainability needs to be considered as well. The use of larger models can sometimes lead to decreased interpretability, and it may be challenging to identify the most influential features that contribute to the classification of a message. Therefore, future research could explore the use of models such as Longformer, which are designed to handle long sequences of text through windowed attention and global attention for the class. This global attention can possibly be leveraged for explainability. Second, with additional computational resources, another potential area of research is to forgo the use of sentence embedders and input messages directly into a transformer model. This approach could potentially improve the performance of the model by better capturing individual sentences rather than relying on message embeddings that are not trained for the specific task. Third, in addition to model improvements, future research could explore additional processing techniques of influential messages, such as clustering. Clustering could be used to group similar messages together, allowing for an analysis of influential messages. This could be useful for easier identification of patterns in influential messages.

### Practical Implications

Engaging with help seekers expressing suicidal thoughts while recognizing they can be better helped elsewhere is important. However, counselors should be mindful of empathetically guiding them toward the appropriate channels. It is important to keep validating their emotions and ensuring they feel supported rather than dismissed. Standardized responses from macros can be beneficial in the right circumstances. If used without having good rapport with the help seekers, they can appear distant. Being transparent with the help seeker about the use of macros is important, as well as ensuring good enough rapport between the help seeker and counselor has been established with personal responses before using standardized responses. Collaborative problem solving and building rapport are proven ways to foster better conversations. Positive reinforcement might be another method that counsellors can employ. Including positive reinforcement more regularly in their responses might be beneficial for helpline conversations.

### Conclusion

This study compares the performance of different models for classifying suicide helpline chat conversations and found that a weighted average model using message embeddings performed the best. This study is unique compared to other studies that aim to gain insight into the quality and effectiveness of suicide prevention helplines. Many studies use questionnaires to evaluate implemented counseling approaches. In this study, we identified influential messages that contributed to better or worse scores on a suicide risk questionnaire through a machine learning approach. This initial application showed that we could extract explanations from the model and identified distinct situations for improvement and deterioration of help seekers’ emotional states.

## Abbreviations

BERT: bidirectional encoder representations from transformers
LIME: local interpretable model-agnostic explanations
LSTM: long short-term memory
RoBERTa: robustly optimized bidirectional encoder representations from transformers pretraining approach
TF-IDF: term frequency–inverse document frequency
